# The Impact of Maternal Nanoplastic and Microplastic Particle Exposure on Mammal’s Offspring

**DOI:** 10.3390/cells13161380

**Published:** 2024-08-20

**Authors:** Hong-Ren Yu, Jiunn-Ming Sheen, Mao-Meng Tiao

**Affiliations:** 1 Graduate Institute of Clinical Medical Sciences, College of Medicine, Chang Gung University, Kaohsiung 833, Taiwan; yuu2004taiwan@yahoo.com.tw (H.-R.Y.); ray.sheen@gmail.com (J.-M.S.); 2 Department of Pediatrics, Kaohsiung Chang Gung Memorial Hospital, Kaohsiung 833, Taiwan; 3 Institute for Translational Research in Biomedicine, Chang Gung Memorial Hospital, Kaohsiung 833, Taiwan

**Keywords:** prenatal, nanoplastics, microplastics, mammal, offspring, health

## Abstract

The issue of environmental nanoplastic (NPl) particle and microplastic (MPl) particle pollution is becoming increasingly severe, significantly impacting ecosystems and biological health. Research shows that NPl/MPl can penetrate the placental barrier and enter the fetus, leading to transgenerational effects. This review integrates the existing literature on the effects of prenatal NPl/MPl exposure on mammalian offspring, focusing particularly on its negative impacts on the central nervous system, liver, intestinal health, reproductive function, and skeletal muscles. The vast majority of previous studies on prenatal NPl/MPl in mammals have used polystyrene material. Future research should explore the effects of other prenatal NPl/MPl materials on offspring to better reflect the realities of the human environment. It is also essential to investigate the potential harm and underlying mechanisms associated with prenatal NPl/MPl exposure to offspring in greater depth. This will aid in developing appropriate prevention and treatment strategies in the future.

## 1. Introduction

Globally, up to 400 million tons of plastic are produced annually, with a recycling rate of only 9% since 2012 [1,2]. A substantial amount of plastic debris already exists in the natural environment, with an estimated 5 trillion plastic pieces weighing over 250,000 tons floating in the world’s oceans as of 2013 [3]. Plastic polymers are classified as permanent organic pollutant substances (POPSs) due to the additives they contain. In fact, plastic production includes many additives such as antioxidants, lubricants, corrosion inhibitors, plasticizers, adhesives, heat stabilizers, and flame retardants [4]. However, in environmental and biological systems, plastics undergo biotic and abiotic weathering and degradation, resulting in the generation of numerous tiny particles. These tiny plastic particles are a serious environmental threat, given their almost universal presence in all ecosystems [5]. Tiny plastic particles are transported over long distances through processes such as wind dispersal, river flow, and ocean currents. Consequently, they are widely distributed in water bodies, soils, and the atmosphere worldwide [6]. They enter the biological cycle of numerous species through ingestion and respiration, potentially harming the health and reproduction of these organisms. This may eventually lead human exposure to these tiny plastic particles via the consumption of contaminated seafood and water [7,8]. Consequently, the presence of tiny plastic particles has become a matter of heightened concern. According to research, each individual may ingest tiny plastic particles between 0.1 to 5.0 g per week, with sizes not exceeding 1 mm, which is equivalent to 74,000 to 121,000 particles per year [9,10]. The majority of ingested plastic particles, especially larger ones, are excreted through feces; however, smaller plastic particles are more likely to be absorbed by the individual [11]. According to ISO 24187:2023 [12], these tiny plastic particles are classified by their size as nanoplastics (NPls) (<1 µm) and microplastics (MPls) (>1 µm and <5 mm) [13]. Smaller particles often exhibit higher toxicity than larger plastic particles at equivalent mass concentrations. Based on accumulated evidence, plastic particles with a diameter of less than 150 μm can penetrate intestinal epithelial cells, while those ranging from 0.1 to 10.0 μm in diameter can cross the blood–brain barrier and biological membranes [14].

NPls/MPls have been detected in numerus human biological samples, including the saliva [15], breast milk [16], lungs [17,18], liver [19], kidneys [20], colon [21], and blood [22]. These findings can substantially expand our understanding of human exposure to NPl/MPl. Given the ongoing challenge in quantifying human exposure to NPl/MPl, assessing their impact on other organisms has become an important method of better understanding the effect of NPl/MPl exposure on the human body [23]. Although numerous studies have explored the effects of NPl/MPl on cells and aquatic animals, relatively few have focused on their impact on terrestrial animals [24]. In cell culture experiments, short-term exposure to high concentrations of polystyrene (PS) particles is commonly used for research purposes. This exposure induces oxidative stress [25,26], inflammatory responses [27], mitochondrial dysfunction [26], lysosomal impairment [28], apoptosis [27], and genetic toxicity [7,29,30]. Upon feeding mice with 5 and 20 µm of PS-MPl for 28 days, PS-MPls were detected in the liver, kidneys, and intestines [31]. Studies have indicated that MPl accumulation in mouse liver tissue can lead to an imbalance in energy and lipid metabolism, an increase in oxidative stress, neurotoxic responses, gut dysbiosis, and impairment of the intestinal barrier [31,32,33,34]. NPls/MPls can also pose health risks by adsorbing other chemical pollutants such as phthalates, bisphenol A, brominated flame retardants, polychlorinated biphenyls, and heavy metals [35,36].

Animal experiments and human placental studies have confirmed that NPls can traverse the placental barrier from mother to fetus through diffusion or by binding with cell transport proteins, reaching multiple tissues of the developing fetus [37,38,39]. In a recent observational study, Ragusa et al. [40] reported the presence of 12 MPl fragments, three of which were identified as stained polypropylene (PP), with only pigments identified for the other nine. These fragments ranged in size from approximately 5 to 10 µm in samples collected from four human placentas, demonstrating a substantially larger particle size than that detected in a previous study [39]. Accordingly, MPls can be transferred from the mother to the unborn fetus and pose a greater risk to offspring than previously understood.

Animal experiments have also revealed that maternal exposure to 0.5 μm NPl or 5 μm MPl can alter the energy and lipid metabolism of offspring, indicating the potential transgenerational effects of NPl/MPl particle exposure [41]. The Developmental Origins of Health and Disease (DOHaD) concept focuses on exploring how exposure to environmental factors during the perinatal period can affect the development of non-communicable diseases in later life, such as diabetes, fatty liver, hypertension, chronic kidney disease, asthma, and cognitive impairment [42]. According to this theory, physiological and structural changes in the fetus due to environmental stress may increase the likelihood of diseases in the future. This influence may involve metabolic processes, immune function, and tissue development, leading to long-term effects on health and potential disease risks. Considering the potential multigenerational adverse impacts and mechanisms of NPl/MPl exposure on biological systems, it is important to investigate the transgenerational effects of prenatal microplastic exposure and explore effective strategies for managing NPl/MPl. To date, most research on NPl/MPl has focused on their impact on the health of adult humans and animals. However, their influence on next-generation development has not been thoroughly studied. The purpose of this review was to summarize the existing literature on the effects of prenatal exposure to NPl/MPl on offspring, particularly focusing on mammals to provide new opportunities for the prevention or treatment of toxic hazards brought about by environmental NPl/MPl contamination.

## 2. Methods

The data collection process involved a systematic literature analysis using two comprehensive databases, PubMed/Google Scholar (accessed on 8 August 2024), and a thorough search strategy. Different keyword combinations were used, including “microplastic”, “nanoplastic”, “prenatal”, “maternal”, “fetus”, and “offspring”, which were combined with one another. Each category was searched independently in the databases. The search focused solely on original articles published in English, which had to be accessible through open access or institutional subscriptions, while excluding review articles, conference abstracts, and book chapters. The initial search yielded 3110 items across all analysis groups, which were subsequently filtered. The filtering process began with a manual review of titles and abstracts to eliminate duplicate entries and exclude irrelevant publications. Irrelevant publications primarily fell into one of the following categories: (1) model studies (in vitro or computational); (2) NPl/MPl analyses in non-mammalian species; (3) studies related to NPl/MPl that involved only the maternal, placenta, or cell lines without reference to the fetus or offspring; (4) publications that only described microplastic tissue deposition without discussing histological or functional impacts; and (5) studies that solely explored the effects of other chemical components, such as plasticizers or bisphenol, A on offspring. After carefully reviewing and selecting the literature, a total of 20 of the most detailed original studies were ultimately included and analyzed in detail.

## 3. The Impact of Prenatal Exposure to NPl/MPl on the Offspring of Mammals

Regarding the effects of plastic particle exposure during pregnancy on the number of live births and birth weight of offspring in pregnant dams, different studies have shown inconsistent results. With PE-MPl administered by gavage to mice, the PE-MP treated group had fewer live births per dam and lighter pups compared to the control group [43]. Another study indicated that prenatal exposure to PS-NPl reduces the birth weight of offspring but does not affect the number or survival rate of the offspring [44,45]. This inconsistency may be related to variations in experimental design and the different species used in the studies. As a result, when choosing articles to read, it is essential to consider the specific details of each study’s experimental design. Next, the effects of prenatal NPl/MPl exposure on different organs will be discussed individually.

### 3.1. The Iimpact of Prenatal NPl/MPl Exposure on the Central NervousSystem and Retina of Mammalian Offspring (Table 1)

Upon ingestion by mammals, NPl/MPl may exert local effects or enter the bloodstream to reach different organs and tissues [46,47]. Regarding maternal–fetal transfer, after maternal ingestion, NPl/MPl can penetrate the placenta and accumulate in fetal tissues, although the precise mechanisms by which MPl and NPl cross the placental barrier remain elusive [38,39,40]. In mice, prenatal and postnatal exposure to PS particles resulted in their accumulation in several brain regions, including the brainstem [48]. This suggests that PS particles can penetrate the blood–brain barrier and accumulate in the brains of offspring. Yang et al. [49] administered PS-NPl (0.1 μm) and PS-MPl (1 μm) orally to pregnant C57BL mice from gestational days 1 to 17 at a dosage of 1 mg/day and observed the effects on fetuses. The authors found that maternal exposure to MPl and NPl during pregnancy can lead to the deposition of both types of particles in the placenta; however, only PS-NPl particles penetrated the fetal hypothalamus. Offspring showed reduced levels of γ-aminobutyric acid in the prefrontal cortex and amygdala before the 8th week and exhibited anxiety-like behavior.

In pregnant and lactating dams, Jeong et al. [38] found that oral exposure of pregnant and lactating dams to PS-NPl resulted in a higher transfer of these particles to the brains of offspring through lactation than through the placenta prenatally. The authors [38] found that transcriptional expression of genes associated with neurogenic stem cell proliferation was reduced in the hippocampal region of offspring mice, accompanied by an imbalance in energy metabolism in neuroglial cells, upon oral exposure of pregnant and lactating mice to PS-NPl. Prenatal and postpartum exposure to PS-NPl substantially reduced the thickness of the neuronal layer and corpus callosum in the CA3 region of the offspring. Moreover, exposure to high concentrations of PS-NPl could result in abnormal brain development, potentially inducing sex-specific neurological abnormalities and cognitive defects in females [38].

Shin et al. [48] administered PS-NPl orally to C57BL/6J dams during pregnancy and lactation. The authors found that PS-NPl exposure suppressed the expression of brain development-related genes (*Gabra2*, *Fgf8*, *Shh*, *Wnt2b*, *Wnt3*, *Ccnd1*, *Ctnnb1*, and *Creb1*) in the embryonic brain and altered the expression of the *Gabra2* gene and protein in the brains of adult offspring mice. Exposure to PS-NPl during pregnancy and lactation led to anxiety- and depression-like behaviors, as well as social deficits in offspring mice. However, it did not affect cognitive function or nest-building behavior in the mouse offspring [48]. These studies were designed to simulate exposure to plastic particles during pregnancy and postpartum without specifically examining the effects of maternal ingestion of PS-NPl solely during pregnancy on the offspring’s brain. In C57BL/6J and CD-1 mice, prenatal polyethylene (PE)-MPl exposure resulted in repetitive and compulsive behaviors, increased social interaction, reduced social novelty, and reduced spatial working memory, indicating the occurrence of autism spectrum disorder-like behaviors [50].

During pregnancy and lactation, administering 0.05 μm PS-NPl via gavage to female SD rats resulted in changes to monoamine neurotransmitters in the cortex and amino acid neurotransmitters in the hippocampus (e.g., downregulation of GABA) in their offspring by the age of 22 days. Histological observations show disorganized cortical migration in the neocortex of 22-day-old offspring, characterized by reduced cortical plate thickness, excessive proliferation of superficial layer neurons, and a decrease in the number of deep-layer neurons. By the seventh week after birth, the offspring also exhibit anxiety behaviors and deficits in spatial memory [51].

In pregnant dams, PS-NPl exposure via the lungs resulted in NPl penetration of the placental barrier, ultimately reaching the fetus. Fournier et al. [37] administered 20 nm-sized PS-NPl (2.64 × 10^14^ particles) via tracheal instillation to Sprague Dawley (SD) rats on gestational day 19. After 24 h, fluorescence microscopy revealed the presence of PS-NPl in the placenta, fetal brain, liver, lungs, heart, and kidneys, indicating that NPl can traverse the placental barrier and cause deposition in fetal tissues upon late gestational maternal lung exposure.

The retina is an extension of the central nervous system that undergoes terminal differentiation, possessing a unique structure and function responsible for capturing light and converting it into neural signals for visual processing. Recent research indicates that maternal exposure to PS-NPl can adversely affect the retinal development and function of offspring mice [52]. Providing maternal mice with drinking water containing 10 mg/L of PS-NPl (0.1 μm) during gestation and lactation led to developmental and functional abnormalities of the neural retina in their offspring. The authors observed the deposition of PS-NPl in the retinal tissues of offspring along with a decrease in the number of retinal ganglion cells and bipolar cells. Furthermore, offspring exposed to prenatal PS-NPl exhibited delayed development of retinal vasculature and abnormal electroretinogram (ERG) responses, coupled with increased oxidative stress levels. At the molecular level, amino acid metabolism dysregulation and alterations in gene expression were noted in the retinal tissues of the exposed offspring. The pathways mediated by the Fos gene may be a potential target affected by PS-NPl exposure during retinal development.

**Table 1:** The impact of prenatal NPl/MPl exposure on the central nervous system of mammalian offspring.

Material	Design	Size	Species/Stage	Effects	Ref.
PS	Prenatal oral ingestion	0.1 μm NPl + 1 μm MPl	C57BL mice PND 8 W	Anxiety-like behavior Reduced GABA level in the prefrontal cortex and amygdala	[49]
PS	Prenatal and postpartum oral ingestion	0.05 μm NPl	C57BL/6J mice PND 1 and 8–10 W	Impact on the function of neural stem cells and the neuronal cells Induced brain dysfunction	[38]
PS	Prenatal and postpartum oral ingestion	0.193 μm MPl and 0.04 μm NPl	C57BL/6J mice PND 16 W	Downregulated the expression of genes related to brain development in the embryonic brain Reduced *Gabra2* expression in both embryonic and adult brains Offspring mice exhibited anxiety-like and depressive behaviors as well as adverse social behavior	[48]
PE	Prenatal oral ingestion	10–20 μm MPl	C57BL/6J and CD-1 mice PND 5–6 W	ASD-like behavioral traits Decreased social interaction, social novelty, and spatial working memory Increased repetitive and compulsive behavior	[50]
PS	Prenatal and lactation oral ingestion	0.05 μm NPl	SD rats PND 3 W (22 day)/7 W	Changes in monoamine neurotransmitters (cortex) and amino acid neurotransmitters (hippocampus) Cortical plate thickness reduced, excessive proliferation of superficial layer neurons, decreased number of deep-layer neurons Anxiety behaviors and deficits in spatial memory (PND 7 W)	[51]
The impact of prenatal NPl/MPl exposure on the retina of mammalian offspring					
PS	Prenatal and lactation oral ingestion	0.1 μm NPl	C57BL/6 mice PND 3 W	PS-NPl deposition in retina Developmental defects in neural retina and vascular retina, abnormal ERG responses, increased oxidative stress Dysregulations in amino acid metabolism and gene expression; Fos-mediated pathway may serve as a key target	[52]

Abbreviations: ASD, autism spectrum disorder; ERG, electroretinogram; GABA, γ-aminobutyric acid; MPl, microplastics; NPl, nanoplastics; PE, polyethylene; PND, postnatal day, PS, polystyrene; SD, Sprague Dawley.

### 3.2. The Impact of Prenatal NPl/MPl Exposure on the Liver and Metabolism of Mammalian Offspring (Table 2)

Luo et al. [41] administered pregnant mice with 0.5 μm of PS-NPl or 5.0 μm of PS-MPl in water at concentrations of 100 or 1000 μg/L. The authors detected the presence of metabolic disruptions in serum levels of triglycerides (TGs), total cholesterol, high-density lipoprotein cholesterol, and low-density lipoprotein cholesterol, as well as in the liver TC and TG levels of 6-week-old offspring, with 5 μm MPl having a greater impact than 0.5 μm particles. Prenatal NPl/MPl exposure can alter the expression of genes involved in fatty acid synthesis in the liver, which may be an underlying mechanism involved in the disruption of fatty acid metabolism [41].

In our literature review, we found that administering a high-fat diet and/or 5 µm PS-MPl to pregnant SD rats increased liver lipid accumulation, as determined by histological examination, in offspring on postnatal day 7 [53]. Simultaneous exposure to a prenatal high-fat diet and prenatal PS-MPl resulted in severe hepatic steatosis in the offspring. Higher prenatal doses of PS-MPl led to shortened small intestines in the offspring. Prenatal exposure to both a high-fat diet and PS-MPl increases hepatic interleukin-6 levels, apoptosis, and oxidative stress in pups. Moreover, simultaneous exposure to a prenatal high-fat diet and prenatal PS-MPl exacerbated hepatic apoptosis and oxidative stress in offspring [53]. These findings suggest that prenatal co-exposure to another adversity may exacerbate the liver damage caused by prenatal microplastic exposure in offspring.

Moreover, PS-MPl exposure during pregnancy and lactation in mice can lead to maternal metabolic disorders, gut microbiota dysbiosis, and intestinal barrier impairment [34]. Additionally, maternal exposure to PS-MPl was shown to induce transgenerational effects, resulting in decreased levels of glucose, acetate, TC, and TG in F1 female descendants; decreased TC levels in the liver; and changes in the mRNA expression of genes related to carbohydrate and lipid metabolism in F1 offspring. Exposure of animals to PS-MPl during pregnancy and lactation can have long-term metabolic consequences in both F1 and F2 generations [34]. The inflammatory response appears to play a pivotal role in these transgenerational effects. Huang et al. [44] found that Kunming mouse offspring exposed to PS-NPl (0.1 μm in size) during the prenatal and lactational stages exhibited a decrease in liver weight, hepatic oxidative stress, inflammatory cell infiltration, enhanced proinflammatory cytokine expression, and disturbed hepatic glycometabolism.

In contrast to oral ingestion, one study evaluated the impact of inhalation exposure to PS-NPl on the development of non-alcoholic fatty liver disease in mothers and offspring [54]. Maternal exposure to high doses of PS-NPl during the gestational stage led to hepatic steatosis in adult female offspring but not in male offspring. Hepatic gene expression related to free fatty acid uptake and TG synthesis in the glycerol 3-phosphate pathway was elevated in female offspring. This study highlighted the potential health hazards associated with inhaled MPl and NPl.

**Table 2:** The impact of prenatal NPl/MPl exposure on the liver and metabolism of mammalian offspring.

Material	Design	Size	Species/Stage	Effects	Ref.
PS	Prenatal oral ingestion	0.5 μm NPl and 5 μm MPl	ICR mice PND 6 W	Fatty acid metabolism dysregulation	[41]
PS	Prenatal oral ingestion	5 μm MPl	SD rats PND 1 W	Liver steatosis, apoptosis, inflammation, ROS increase, and villi atrophy Combination with a high-fat diet during pregnancy may exacerbate certain pathologic changes	[53]
PS	Prenatal intra-tracheal ingestion	0.02 μm NPl	SD rats Fetus	NPls were detected in the placenta, fetal liver, lungs, heart, and kidneys.	[37]
PS	Prenatal and postpartum oral ingestion	0.1 μm NPl	Kunming mice PND 3 W & 8 W	Liver weight ↓, hepatic ROS ↑, inflammatory cell infiltration ↑, and proinflammatory cytokine expression ↑ Disturbance in hepatic glycometabolism	[44]
PS	Prenatal and postpartum oral ingestion	5 μm MPl	ICR mice F1 generation at PND 6 W and PND 40 W. The F2 generation at PND 6 W	Reduced levels of glucose, pyruvate, total cholesterol and triglyceride in F1 female offspring Altered hepatic cholesterol in F1 generation offspring Alteration in mRNA expressions of gene related to glycolipid metabolism Changes in the gut microbiome	[34]
PS	Prenatal inhalation	≈0.07 μm NPl	C57BL mice PND 12 W	Hepatic steatosis in adult female offspring but not male offspring Elevated expression of genes related to fatty acid uptake and tri-glycerol synthesis in the G3P pathway	[54]

Abbreviation: G3P, glycerol 3-phosphate; MPl, microplastics; NPls, nanoplastics; PND, postnatal day; PS, polystyrene; ROS, reactive oxygen species; SD, Sprague Dawley.

### 3.3. The Effects of Prenatal NPl/MPl Exposure on the Intestines of Mammalian Offspring (Table 3)

Prenatal MPl-exposure-induced gut damage has been characterized. In an SD rat model, prenatal PS-MPl exposure decreased ileum length [53]. Additionally, another study revealed the occurrence of histological changes in the small intestine, increased reactive oxygen species (ROS), and decreased abundance of glutathione peroxidase 4 (GPx4), ferritin heavy chain 1 (FTH1), and ferritin light chain (FTL) proteins, indicating the initiation of ferroptosis [55].

**Table 3:** The effects of prenatal NPl/MPl exposure on the intestines of mammalian offspring.

Material	Design	Size	Species/Stage	Effects	Ref.
PS	Prenatal oral ingestion	0.08 μm NPl	C57BL/6J mice	Histological changes in small intestine Upregulation of ROS Downregulation of GPx4, FTH1, and FTL protein levels, indicating initiation of ferroptosis	[55]
PS	Prenatal oral ingestion	5 μm MPl	SD rats PND 1 W	Decrease in villi length of offspring ileum upon high prenatal MPl exposure	[53]

Abbreviation: FTL, ferritin light chain; FTH1, ferritin heavy chain 1; GPx4, glutathione peroxidase 4; MPl, microplastics; NPl, nanoplastics; PND, postnatal day; PS, polystyrene; ROS, reactive oxygen species; SD, Sprague Dawley.

### 3.4. The Effects of Prenatal NPl/MPl Exposure on the Reproductive System of Mammalian Offspring (Table 4)

There have been many studies and reviews on the reproductive toxicity of NPl/MPl in mammals, revealing that NPl/MPl [56,57,58] can induce reproductive toxicity through various mechanisms. However, most of these studies have been conducted using adult rodents, with less exploration of the effects during early developmental stages. Upon exploring the toxic effects of pre- and postnatal exposure to NPl on testicular development and reproductive function, one study reported that exposure to 0.5 μm PS-NPl pre- and postnatally leads to testicular dysplasia in offspring at day 35 (pre-puberty stage) and sperm production dysfunction at day 70 (sexual maturity stage) after birth [59]. Zhao et al. [59] administered drinking water containing varying concentrations (0.5, 5, and 50 mg/L) of PS-NPl to pregnant mice and their offspring from gestational day 1 to postnatal day (PND) 35 or PND 70. The authors found that exposure to 5 and 50 mg/L NPl delayed the onset of puberty in the offspring at PND 35. Transcriptome analysis of immature testes revealed the hormone-mediated signaling pathway, G1/S transition of the mitotic cell cycle, coregulation of androgen receptor activity, and the Hippo signaling pathway were involved upon exposure to PS-NPl [59]. In a study conducted by Huang et al. [44], pre- and postnatal PS-NPl exposure led to decreased testicular weight, seminiferous epithelium disruption, and decreased sperm count in mouse offspring. Testicular damage induced by prenatal and postnatal PS-NPl exposure was associated with oxidative injury [44].

Regarding the toxic effects of perinatal PE-MPl exposure on female reproduction in offspring, a recent study showed that perinatal (prenatal and lactation stage) PE-MPl exposure reduced oocyte maturation, fertilization rate, and embryo development in female offspring [60]. Despite the lack of a specific focus on prenatal exposure, these studies revealed that perinatal exposure to NPl/MPl could exert toxic effects on the reproductive systems of both males and females.

Recently, Dou et al. published an important study. The authors fed lactating female mice with PS-MPl at concentrations corresponding to those detected in baby formula prepared using plastic bottles [61]. The results led to reproductive toxicity in F0 female mice, manifested as delayed puberty, disturbed estrous cyclicity, decreased fertility, elevated testosterone levels, abnormal follicle development, and autoimmune ovarian inflammation. Notably, the experimental results showed that F1 male offspring exhibited decreased sperm count, reduced sperm motility, and altered testicular gene expression profiles. Furthermore, F2 male offspring also showed a pattern of reduced sperm count. These results indicate that male offspring are significantly more susceptible to intergenerational and transgenerational reproductive toxicity caused by maternal exposure to PS-MPl compared to female offspring.

**Table 4:** The effects of prenatal NPl/MPl exposure on the reproductive system of mammalian offspring.

Material	Design	Size	Species/Stage	Effects	Ref.
PS	Prenatal and postpartum oral ingestion	0.5 μm NPl	ICR mice PND 5 W & 10 W	Testicular development and sperm production impacted through the Hippo signaling pathway and an imbalance in the immune microenvironment in F1 male offspring	[59]
PS	Prenatal and postpartum oral ingestion	0.1 μm NPl	Kunming mice PND 3 W & 8 W	F1 male offspring showed a reduction in testicular weight, disruption in the seminiferous epithelium, and decreased sperm count	[44]
PE	Prenatal and postpartum oral ingestion	10–150 μm MPl	Kunming mice PND 8 W	F1 female offspring showed a reduction in the oocyte maturity, fertilization rate, and embryo development	[60]
PS	F0 exposure at lactational stage	1 μm MPl	ICR mice	Epididymal semen concentration and sperm viability decreased in F1 male offspring Downward trend in sperm counts in F2 male offspring	[61]

Abbreviation: MPl, microplastics; NPl, nanoplastics; PE, polyethylene; PND, postnatal day; PS, polystyrene.

### 3.5. The Effects of Prenatal NPl Exposure on the Skeletal Muscle System of Mammalian Offspring (Table 5)

The skeletal muscle is a metabolically active organ that communicates with other organs through the secretion of proteins to regulate energy metabolism. Chen et al. [45] investigated the effects of maternal PS-NPl exposure during pregnancy using transcriptomic and metabolomic analyses. Maternal PS-NPl exposure (0.1 μm; 10 mg/L) via drinking water led to substantial gene dysregulation in cholesterol and lipid metabolism, muscle tissue development, and skin formation in the muscle tissue. This study offers new insights into fetal effects in mice exposed to prenatal PS-NPl.

**Table 5:** The impact of prenatal NPl/MPl exposure on the skeletal muscle of mammalian offspring.

Material	Design	Size	Species/Stage	Effects	Ref.
PS	Prenatal oral ingestion	0.1 μm NPl	C57BL/6J fetus	Dysregulated expression of genes regulating cholesterol and lipid metabolism, muscle tissue development, and skin formation	[45]

Abbreviation: NPl, nanoplastics; PS, polystyrene.

### 3.6. Limitations and Future Research Directions

Based on the accumulated literature, five main types of MPl have been detected in the human body: general polyester (39%), polyamide (17%), polyurethane (15%), polypropylene (9%), polyacrylate (8%), and PS (2%) [62]. Among these materials, polystyrene (PS) is widely used in packaging, food storage, and textiles due to its excellent thermal stability and moldability [61]. It is also considered the most representative source of environmental emissions. Therefore, PS-NPls/MPls have often been selected as research materials to explore the potential toxicity of NPl/MPl in animals. In the past, nearly 80% of the research focused on the toxicological effects of single-sized PS spheres on animals [62]. These studies provide evidence regarding the effects of specific types and characteristics of NPls or MPls on animals. Nevertheless, studies on the toxicity of different microplastic polymer types in rodents remain limited. Therefore, additional investigations are needed to explore the impact of NPl/MPl combinations by simulating exposure in organisms as closely as possible to real-world environmental conditions. The generated evidence would facilitate the establishment of more reliable and thorough conclusions regarding their potential health risks.

Due to the challenges involved in detecting NPl/MPl exposure in humans, there are currently only a limited number of human studies that explore NPl/MPl in reproductive tissues and assess their correlation with fertility and pregnancy outcomes. A recent study demonstrated a negative correlation between the accumulation of MPl in the placenta and neonatal birth weight, length, head circumference, and 1 min APGAR scores [63]. Additionally, reports have suggested a negative correlation between the concentration of MPl found in amniotic fluid collected at delivery and gestational age, indicating that MPl exposure during pregnancy may lead to preterm birth [64]. However, these studies generally have small sample sizes, and data on the prevalence of NPl/MPl in humans and the placenta remain insufficient, highlighting the gaps in current research. Identifying prevention and management strategies based on research findings is another urgent priority.

## 4. Conclusions

**Figure 1:**
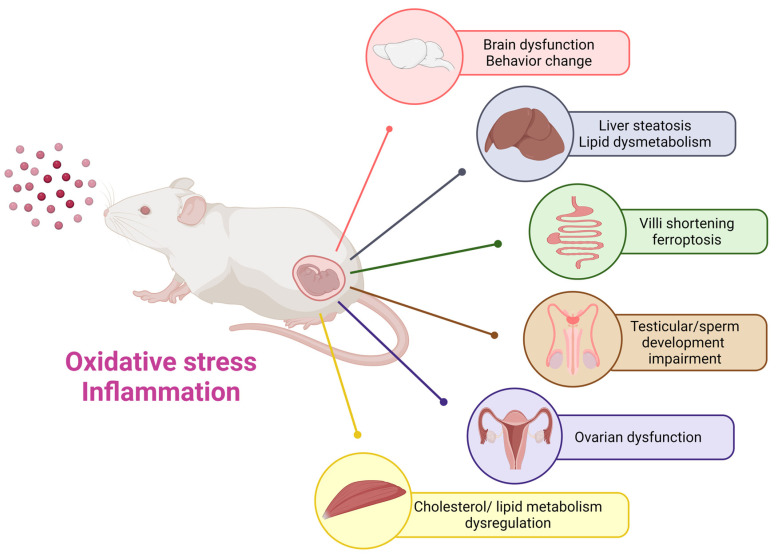
The impact of prenatal microplastic exposure on various organs of the offspring, including the brain, liver, intestine, reproductive system, and skeletal muscle. This diagram emphasizes the potential risks linked to maternal exposure to nanoplastics and microplastics, highlighting the necessity of further research to comprehensively clarify long-term effects on the health of offspring.

NPls/MPls have already invaded our lives and may impact the health of future generations. During the perinatal period, embryos and individual organs develop rapidly and become extremely sensitive. Existing research suggests that prenatal exposure to NPl/MPl can adversely affect the central nervous system, liver, intestine, reproductive system, and skeletal muscles of mammalian offspring through oxidative stress, inflammatory and other mechanisms (Figure 1). Previous research on prenatal NPl and MPl in mammals has predominantly focused on polystyrene materials. To more accurately represent the realities of the human environment, future studies should examine the effects of other prenatal microplastic materials on offspring. Moreover, it is crucial to delve deeper into the potential harms and mechanisms linked to prenatal NPl/MPl exposure in offspring. This research will aid in formulating effective prevention and treatment strategies moving forward.

## Data Availability

No new data were created or analyzed in this study. Data sharing is not applicable to this article.
